# Rare and Costly Prosocial Behaviors Are Perceived as Heroic

**DOI:** 10.3389/fpsyg.2019.00234

**Published:** 2019-02-05

**Authors:** Gordon T. Kraft-Todd, David G. Rand

**Affiliations:** ^1^Department of Psychology, Yale University, New Haven, CT, United States; ^2^Management Science, Brain and Cognitive Sciences, Massachusetts Institute of Technology, Cambridge, MA, United States

**Keywords:** heroism, cooperation, social norms, prosocial behavior, altruism

## Abstract

Heroism has only recently become a topic of empirical investigation. Existing research suggests a connection between heroism and four well-documented dimensions of human social behavior: (1) the cost incurred by the actor; (2) the benefit provided to the recipient; (3) the perceived frequency (i.e., descriptive normativity); and (4) the perceived expectation to perform it (i.e., injunctive normativity). In a series of exploratory studies (total *N* = 408), we aim to shed light on how each of these constructs influence lay intuitions about the nature of heroism (i.e., what determines which acts people perceive to be heroic). In Study 1, subjects generated a list of acts they deemed to be heroic. In Study 2, subjects rated the heroicness of the acts from Study 1, revealing considerable variation in the level of heroism. Finally, subjects in Study 3 rated the cost to the actor, the benefit to the recipient(s), the descriptive normativity (i.e., frequency), and the injunctive normativity (i.e., obligatoriness) of ten acts, five of which received particularly high heroism scores in Study 2 (“exemplary” acts of heroism) and five of which received particularly low heroism scores in Study 2 (“ambiguous” acts of heroism). We find that more heroic acts are seen as rarer and more costly to actors—but, interestingly, *not* more beneficial to recipients or less obligatory. These findings help to illuminate what it means to be seen as a hero, and suggest clear future directions for both empirical and theoretical work.

## Introduction

Heroism is the original topic of literature, as evidenced by some of the earliest known human writing from approximately 2100 BC in the Epic of Gilgamesh. Yet, an empirical understanding of heroism is only just emerging. This research has variously investigated types of heroism (Franco et al., 2011), functions of heroism (Kinsella et al., 2015a), traits of heroes (Goethals and Allison, 2012), characteristics of heroes (Kinsella et al., 2015b), and gender differences among heroes (Becker and Eagly, 2004), as well as amalgams of these approaches (Riches, 2018). Allison et al. (2016) summarize a number of dichotomies made in the literature which try to distinguish between two classes of heroes (e.g., emergent vs. sustained; Kraft-Todd and Rand, 2016). In light of this research, an early consensus definition of heroism seems to be *taking extraordinary action in service of the greater good with personal risk of significant sacrifice* (Allison et al., 2016).

Thus articulated, the burgeoning science of heroism appears to sit squarely between two social science literatures: game theory and social norms. Game theory formalizes strategic decision-making between individuals by quantifying the costs and benefits at stake in an interaction (Von Neumann, 1959). This conceptualization allows for a precise definition of *cooperation*—an individual paying a cost to give another a benefit—which in turn presents a conundrum: why do people cooperate (Rand and Nowak, 2013)? A particularly challenging problem in game theoretic terms is understanding why an individual would pay a cost to give *many others* a benefit, i.e., contribute to public goods (Hardin, 1968)—a pressing problem shared by policy-makers in the real world (Kraft-Todd et al., 2015). In the language of game theory, then, the risk of sacrifice in heroism implies a potentially large cost paid by an actor in order to cooperate or contribute to the public good. Heroism thus may be understood as a special case of cooperation in which the actor incurs (or at least risks) a large cost (akin to extreme altruism; Marsh et al., 2014; Rand and Epstein, 2014). Further, there is good reason to believe that assessment of the costs and benefits might be relevant to our perception of heroism. Adults and children use information about the costs and benefits of others’ behaviors to make inferences about their character (Jara-Ettinger et al., 2016). As early as 2 years of age, these evaluations affect our preferences for interacting with others (Jara-Ettinger et al., 2015). Thus, ascriptions of heroism may rely on beliefs about the costs and benefits of an actor’s behavior.

Social norms are “rules and standards that are understood by member of a group, and that guide and/or constrain social behavior without the force of laws” (Cialdini and Trost, 1998). Two types of social norms are frequently distinguished: *descriptive* norms, which are about what people think others do; and *injunctive* norms, which are about what people believe others think they *should* do. Colloquially, our conception of what is “normal” lies somewhere between our conception of what is descriptively and injunctively normative (Bear and Knobe, 2016). In the language of social norms, then, the extraordinary action that defines heroism is descriptively *non*-normative (i.e., rare).

Conceptually situated within this overlap of game theory and social norms, four quantifiable dimensions of social perception may help elucidate a clearer empirical understanding of heroic behavior: (1) the cost to the actor; (2) the benefit to the recipient(s); (3) the descriptive normativity of the behavior; and (4) the injunctive normativity of the behavior. In a series of exploratory studies (total *N* = 408), we aim to discover the extent to which these constructs influence people’s perceptions (i.e., lay intuitions) of heroism.

Intuitively, it seems likely that the more a behavior is thought to be heroic, the greater would be the perceived cost to the actor and benefit to the recipient, while the lower would be the descriptive and injunctive normativity of the behavior. We use a “ground-up” approach to the concept of heroism, avoiding *a priori* assumptions about what “counts” as heroism (similar to the method of Kinsella et al., 2015a). In Study 1, we therefore ask subjects to generate acts of heroism. In Study 2, we ask a separate group of subjects to rate the extent to which these candidate behaviors are heroic. Finally, in Study 3, we ask yet another group of subjects to rate the extent to which a subset of these candidate behaviors are costly to the actor, beneficial to the recipient, descriptively normative, and injunctively normative.

## Study 1: Subject-Generated Acts of Heroism

### Materials and Methods

We recruited 102 subjects from the online labor market Amazon Mechanical Turk (mTurk; Horton et al., 2011; Arechar et al., 2017). We did not collect standard demographics such as age and gender, though previous research has shown that this population is more representative than typical student samples (Berinsky et al., 2012), if not representative of the national population (Paolacci and Chandler, 2014). Subjects completed the study in *m* = 5 min and were paid $0.50 for their participation, commensurate with typical rates on this platform. We prevented subjects from participating repeatedly (both within each study and across studies) by excluding duplicate Amazon worker IDs and IP addresses. Our pre-study procedure (in this and following studies) was to ask subjects to provide their mTurk IDs and transcribe a sentence of difficult-to-read handwritten text (the latter to prevent bot participation and discourage low-effort workers). For Study 1, subjects simply responded to the prompt: “Please name at least 3 and up to 10 real-life acts of heroism” using free-response text boxes.

Data analysis for all studies was completed using STATA 13. Informed and written consent in all studies was obtained from all subjects and was approved by Yale University’s Institutional Review Boards protocol 1307012383.

### Results and Discussion

Subjects generated on average *m* = 4.2 responses, which were edited for responses which did not answer the question (often because they were the wrong part of speech, e.g., “boldness,” “Jon Meis”) repeated answers (within subjects), spelling, punctuation, and grammar (see Supplementary Table S1 for complete list of unedited responses). Responses were further edited for simplicity (e.g., generalizing pronouns such as “woman” and “man” to “person”) and semantic commonality (“Entering a burning building to save some one” and “Going into a burning building to rescue people”), yielding a list of 80 unique responses (see Table 1). It is worth noting that nearly all behaviors are explicitly prosocial in nature (e.g., contain “saving,” “rescuing,” “donating,” and “protecting,” etc.).

**Table 1:** Edited list of all candidate acts of heroism used as stimuli in Study 2 (80 total).

Edited behaviors	
A child standing up for another child being bullied	Helping a choking victim (e.g., the heimlich maneuver)
A dog fighting off a wild animal to safe his or her owner	Helping wounded people in a terrorist attack
A person helping their wife deliver their child	Intervening to prevent a rape
A person jumping on a grenade to save fellow soldiers	Jumping onto subway tracks to lift a person to safety
A person shielding someone during a shooting	Performing a life-saving surgery
Admitting mistakes	Playing it forward (e.g., buying someone else a coffee unprompted)
Adopting an animal	Preventing someone from committing suicide
Adopting and raising foster children	Protecting people in immediate danger
Assisting the elderly	Pulling a person from beneath a collapsed wall
Becoming a rescue worker	Pulling people out of a train wreck
Being a first responder in a natural disaster	Pulling someone out of a burning car
Being a good parent	Pushing someone out of the way of an oncoming car
Being a really good friend for someone with depression	Putting out a fire
Being in a search party	Raising your child well
Bringing food or medicine to the elderly or disabled	Reporting a crime to the police
Bringing someone food	Rescuing someone from a flood
Cancer patients fighting for their lives	Sacrificing yourself so strangers may live
Childbirth	Sacrificing yourself so your children may live
Climbing a tree to rescue a pet	Sacrificing yourself so your family may live
Confronting a gunman to defend others	Saving a child from being kidnapped
Confronting an abusive spouse	Saving a dog from a hot car
Conscientious objectors who refuse to go to war	Saving hostages
Covering your loved ones with your body as a tornado hits your home	Saving someone from drowning
Defending someone from abusive authority figures	Saving someone’s life
Defending someone from harm	Saving someone’s life when it is not your job
Donating an organ	Saving someone’s life when it is your job (e.g., fireman, emergency room doctor)
Donating blood	Utility workers restoring power in the middle of a major storm
Donating bone marrow	Volunteering
Donating clothes, toys, or other consumer goods (not food or money)	Volunteering at a soup kitchen
Donating food	Volunteering at an animal shelter
Donating to charity	Whistle-blowing (i.e., reporting wrong-doings in the organization that you work in)
Dying in the line of fire (in the military)	Working as a doctor
Entering a burning building to save someone	Working as a firefighter
Fighting for your country	Working as a nurse
Fighting wildfires	Working as a policeman
Finding a murder suspect	Working as an inner-city school teacher
Giving CPR to a person that needs it	Working for a charity
Giving someone an interest-free loan when they are poor	Working for a non-profit
Going out on the ice to rescue a person who went through the ice	Working in the coast guard
Going to a protest against injustice	Working in the military


*Raw responses from Study 1 (428 total; see Supplementary Table S1) were edited according to which did not answer the question, repeated answers (within subjects), spelling, punctuation, grammar, simplicity, and semantic commonality.*

Study 1 therefore provided us with a list of potentially heroic behaviors. The purpose of Study 2, then, was to assess lay intuitions about how heroic each of these behaviors is perceived to be.

## Study 2: Validating Candidate Acts of Heroism

### Materials and Methods

We recruited 205 subjects from mTurk who did not participate in Study 1. Subjects completed the study in *m* = 3 min and were paid $0.50 for their participation. Following the same pre-study procedure as in Study 1, subjects rated a randomly selected subset of 20 candidate acts of heroism from the 80 generated in Study 1 (presented in randomized order) on how heroic they were using two scales (also presented in randomized order): a binary measure of whether the candidate behavior qualified as “Heroic” (1) or “Not heroic” (0), and a continuous measure of the extent to which the candidate behavior was heroic (Likert scale, 1: “Not at all heroic” – 7: “Very heroic”). Thus *m* = 51 subjects rated each candidate behavior using both of these scales. These measures were strongly and significantly correlated (*r* = 0.95, *p* < 0.001), so we use the binary measure for ease of exposition, though analyses are robust to using either measure (see Supplementary Figure S3 for results of Study 3 using the continuous measure).

### Results and Discussion

**FIGURE 1:**
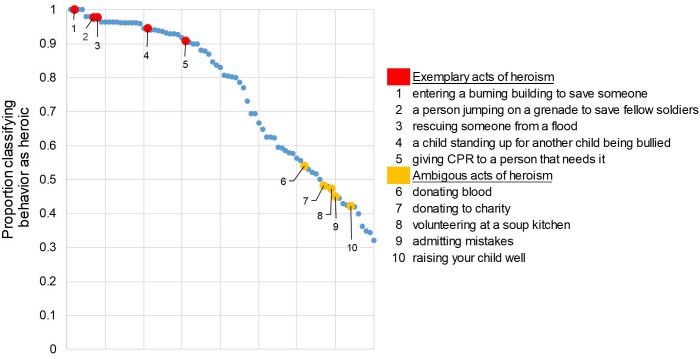
Candidate acts of heroism generated by subjects were broadly (examples in red) or ambiguously (examples in yellow) considered heroic by a separate sample. Shown is a scatterplot of the proportion of subjects rating each of 80 candidate behaviors as “Heroic” (1) or “Not heroic” (0) ranked from most to least heroic. (X-axis represents dummy values for act of heroism).

Across all 80 candidate behaviors, the median percentage of subjects classifying the behaviors as “heroic” was 82% (*m* = 75%; see Figure 1). Thus, subjects from Study 1 appear to have done a satisfactory job of nominating candidate acts of heroism. Critically, however, there was also substantial variation across behaviors in their *level* of heroism.

The goal of Study 3, then, was to understand what explains this variation in heroicness. To do so, we selected ten acts of heroism to investigate in more detail (see Figure 1 legend). We selected five “exemplary” acts of heroism that demonstrated wide consensus on being perceived as heroic (proportion classifying behavior as heroic >0.9) and five “ambiguous” acts of heroism that were not strongly perceived as heroic or not heroic (proportion classifying behavior as heroic = 0.4–0.6). In selecting these behaviors, we focused on behaviors that were frequently discussed in the contexts of cooperation, prosociality, and heroism; and that were specific, rather than sustained, behaviors (with the exception of “raising your child well”).

## Study 3: What Distinguishes Acts Perceived as More Heroic?

### Materials and Methods

We recruited 101 subjects from mTurk who completed the study in *m* = 5 min and were paid $0.50 for their participation. Following the same pre-study procedure as in Study 1, subjects rated each of the 5 “exemplary” and each of the 5 “ambiguous” heroism behaviors on *descriptive normativity* (“In your opinion, how many people in your community do this behavior?”), *injunctive normativity* (“In your opinion, how much do people in your community think doing this behavior is what you are supposed to do?”), *benefit to the recipient* (“In your opinion, how much benefit (in terms of money, time, effort, etc.) does the recipient of this behavior receive?”), and *cost to the actor* (“In your opinion, how much cost (in terms of money, time, effort, etc.) does the person who does this behavior incur?”). The 10 behaviors were presented in randomized order and ratings (also presented in randomized order) were completed using sliding scales which ranged from 0 “Very little” to 100 “Very much.”

### Results and Discussion

First, we investigate the pairwise correlations among our independent variables (Table 2; Pearson’s correlation coefficient, *p*-values Bonferroni corrected for 6 simultaneous comparisons). Though we observe many significant correlations, they are sufficiently low that it is reasonable to investigate the relationship between heroicness and all independent variables simultaneously in a single model.

**Table 2:** Our dependent variables are significantly, though weakly correlated.

	Descriptive normativity	Injunctive normativity	Benefit	Cost
Descriptive normativity	X			
Injunctive normativity	0.45^∗∗∗^	X		
Benefit	0.09	0.24^∗∗∗^	X	
Cost	-0.02	0.09	0.27^∗∗∗^	X


*^∗∗∗^p < 0.001, ^∗∗^p < 0.01, ^∗^p < 0.05. p-values Bonferroni corrected for 6 simultaneous comparisons.*

**FIGURE 2:**
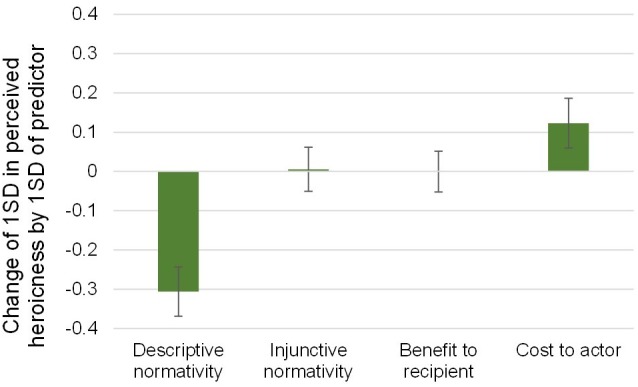
More heroic acts are seen as *rarer* and *more costly* to actors (though *not* more beneficial to recipients or less obligatory) than ambiguous acts of heroism. Shown are standardized coefficients (with 95% CIs) of subjects’ ratings on four measures (0–100 slider scales) predicting the heroicness of the acts as measured on a binary scale (from Study 2).

Therefore, we investigate differences in perceived heroism based on these four dimensions using OLS regression with proportion of Study 2 participants indicating the behavior was heroic (standardized) as the dependent variable, and (standardized) ratings of costliness, benefit, descriptive normativity, and injunctive normativity as independent variables, clustering standard errors on subject (regression coefficients plotted in Figure 2; see Supplementary Figure S1 for a plot of raw means and Supplementary Figure S2 for distributions). More heroic acts were perceived as less descriptively normative [*b* = -0.31, 95% CI (-0.37,-0.24), *t*(101) = -9.64, *p* < 0.001] and more costly to the actor [*b* = 0.12, 95% CI (0.06,0.19), *t*(101) = 3.85, *p* < 0.001]. However, the heroicness of the acts was not significantly related to perceived injunctive normativity [*b* = 0.01, 95% CI (-0.05,0.06), *t*(101) = 0.20, *p* = 0.843] nor perceived benefit to the recipient [*b* = -3.27e-4, 95% CI (-0.05,0.05), *t*(101) = -0.01, *p* = 0.990]. These results are robust to Bonferroni correction for four simultaneous comparisons (i.e., all significant *p*-values are smaller than 0.0125). Because the continuous ratings of heroicness (our dependent measure) are bimodally distributed (by design), we also demonstrate robustness to conducting this analysis using a logistic regression predicting a categorical dependent variable (exemplary vs. ambiguous acts of heroism; see Supplementary Table S2).

## General Discussion

Situating an empirical approach to heroism within the game theory and social norms literatures, we conducted three exploratory studies (total *N* = 408) on lay intuitions of heroic acts. We find that acts which are widely agreed upon as being heroic (exemplary heroism) can be distinguished from acts whose heroicness is unclear (ambiguous heroism), with exemplary acts having lower descriptive normativity and higher costliness to the actor—but *not* differential injunctive normativity nor benefit to the recipient. These results extend prior work on heroism by providing empirical evidence supporting the conceptual link between the emerging science of heroism and more established fields in the social sciences, while also clarifying the lay definition of heroism.

Our approach avoids being bound by academic preconceptions of heroism by utilizing subject-generated acts of heroism as our stimuli (Study 1, in which we asked, “Please name at least 3 and up to 10 real-life acts of heroism”) in subsequent studies. Though a few empirical studies of heroism employ this method, these investigate other aspects of heroism: e.g., “In your view, what functions do heroes serve?” (Kinsella et al., 2015a) and “What are the features that you associate with heroes and their heroic actions?” (Kinsella et al., 2015b). We believe a comprehensive understanding of heroism will be achieved by exploring various means of eliciting lay perceptions of heroism and finding consensus among them.

One major limitation to our investigation is the extent to which our results depend on the specific ten behaviors from Study 2 we chose to serve as stimuli in Study 3. Future research should test the robustness of our conclusions to the consideration of a wider range of heroic acts, and perhaps conduct additional pretesting to ensure candidate acts of heroism do not differ on dimensions (e.g., familiarity) which might affect variables of interest. In particular, the inclusion of candidate acts of heroism with intermediate values of the proportion of people considering them heroic (compared to relative extremes we investigate here)—and the inclusion of acts considered distinctly *non*-heroic—may shed further light on the relation of perceived heroism and our variables of interest.

Additionally, because our results are drawn from a convenience sample of mTurk workers, they may be culturally specific (in accordance with the WEIRD hypothesis; Henrich et al., 2010). Given that heroism is a socially designated role, it is reasonable to think that lay perceptions of heroism would be influenced by cultural norms; for these reasons, future work might seek to assess this phenomenon cross-culturally. It is worth noting that our investigation is on lay perceptions of heroism, rather than the decision-making process of heroes, and so future work might also explore the extent to which the variables we consider are relevant this process, or whether they are the result of more generalized intuitions (Glanville and Paxton, 2007; Rand and Epstein, 2014). One other potentially interesting question for future research would be to compare first-order beliefs about heroism (as we do here) with second-order beliefs (i.e., what subjects think others think about heroism), as the latter have been demonstrated to have a greater impact on prosocial behavior than the former (Jachimowicz et al., 2018). Finally, our investigation was exploratory, so replication and confirmatory studies should be conducted to provide greater faith in our findings and their interpretation.

We found the observation that exemplary acts of heroism were *not* perceived as more beneficial to recipients than ambiguous acts of heroism to be surprising, given our intuition that helping others is part what makes an act heroic [e.g., “a person jumping on a grenade to save fellow soldiers” *m* = 69.08 95% CI (62.51, 75.65) vs. “volunteering at a soup kitchen” *m* = 65.84, 95% CI (60.53, 71.16), *t*(200) = 0.76, *p* = 0.45]. The within-subjects design of Study 3 grants additional credence to this observation: each subject rated all ten of the heroic acts, and so presumably they could have compared one situation to the next and made these judgments relative to each other. Yet, the finding interestingly coincides with other empirical findings. For example, subjects participating in prisoner’s dilemmas are highly influenced by the framing of the situation and potential behaviors, beyond the simple material outcomes of the interaction (e.g., Zhong et al., 2007), and so too here might verbal associations weigh more heavily than numerical assessments in subjects’ judgments. In charitable giving, the “effective altruism” movement aims to direct giving toward more socially efficient causes—i.e., get more bang for the donor’s buck—yet effectiveness information often does not motivate greater giving (Berman et al., 2018). This finding is an example of the broader phenomenon of *scope insensitivity* (Carson, 1997), in which people do not exhibit greater valuation for increased amounts of an economic good. Scope insensitivity has been repeatedly demonstrated in the domain of prosociality (Desvousges et al., 1993; Hsee and Rottenstreich, 2004; Small et al., 2007). Thus, our findings are in a sense the converse: while previous research has shown that people do not value (via monetary donations) causes which provide a greater benefit to others, we show that people do not perceive a greater benefit to others from behaviors that are more valued (via judgments of heroism). Further, and more relevant to the characterological judgment nature of heroism, this (non)relation of social benefit to valuation is consistent with findings that people *do not* prefer consequentialist agents who are willing to inflict harm to provide a greater social benefit (Everett et al., 2018). Taken together, these many potential reasons for our surprising finding that exemplary acts of heroism were not perceived as more beneficial to recipients than ambiguous acts of heroism suggest a promising avenue for future research.

Our finding that judgments of heroism are linked to the cost to the actor but not the benefit to the recipient suggests numerous questions regarding the proximate mechanism of heroism perception. First, when a decision-maker is attempting to distinguish whether another’s behavior is heroic or not, it could be that the costs to the actor are more salient than the benefits to the recipient if this judgment is accomplished via imagining what it is like to be in the actor’s shoes (rather than the recipient’s). For example, it could be that when you are trying to decide whether “entering a burning building to save someone” is heroic or not, you engage in perspective-taking not with the person who might be saved, but with the person entering the burning building. Second, it could be that the costs of heroism are simply more observable than the benefits because calculating the latter requires an extra step of contrapositive reasoning: i.e., it requires knowing *what would have happened if the hero had not intervened*. For example, when a child stands up for another child being bullied, we know that child steps in the way of the bully’s fists, but we don’t know whether the bully would have broken the victim’s nose or just taken their lunch money.

Our finding that judgments of heroism are linked to the *descriptive* normativity of the action but not the *injunctive* normativity was also surprising to us, as our intuition was that “going above and beyond” was an important part of being seen as heroic. Our data indicate, however, that this is not the case. Many of the proposed acts of heroism in Study 1 included professions where taking risks to help others is part of the job expectations (e.g., military, firefighter; see Table 1 and Supplementary Table S1). Thus, for these people acting heroically may not be unexpected (i.e., is injunctively normative), but it still may be *rare* (i.e., is descriptively non-normative). The fact that such actions were still judged to be heroic indicates that unexpectedness (or injunctive normativity) does not appear to be a crucial component of lay perceptions of heroism.

Heroism, understood as rare (i.e., non-normative) and costly cooperation is a particularly timely concept to understand as the need to promote innovative solutions to global social challenges becomes increasingly clear (Kraft-Todd et al., 2018). We hope our conceptualization of heroism can help connect the emerging science to such pressing real-world issues. Heroism needn’t be confined to our cultural mythologies (Campbell, 1949/2008); we may find that we can cultivate it more effectively if we celebrate it in our science as well as in our stories.

## Data Availability Statement

All data are publicly available at: https://osf.io/be8mn/.

## Author Contributions

GK-T and DR designed the online experiments. GK-T conducted the online experiments and analyzed the results. GK-T and DR wrote the manuscript.

## Conflict of Interest Statement

The authors declare that the research was conducted in the absence of any commercial or financial relationships that could be construed as a potential conflict of interest.
