# Supplementary material for: Rare and Costly Prosocial Behaviors Are Perceived as Heroic
Source: Front Psychol. 2019 Feb 5;10:234. doi: 10.3389/fpsyg.2019.00234 (PMC6370665; doi:10.3389/fpsyg.2019.00234)

**SI Table 1. Raw responses of candidate acts of heroism from Study 1 (428 total).**

| A ballet dancer jumped onto subway tracks to lift a man to safety. | going to get your fellow soldier who is wounded under enemy fire | Rushing into a fire |
| --- | --- | --- |
| A boy is pulled from beneath a collapsed wall at the Plaza Towers Elementary School | Great teachers who could have been anything but chose to dedicate their lives to the future | Sacrifice |
| a dog fighting off a wild animal to safe his or her owner | Harvey Randolph | sacrifice ones self |
| A French philosopher who praised risk-taking died while saving drowning children. | Harvey Randolph | Sacrificing for your children. |
| A homeless man aided children wounded in a terrorist attack in England. | Having courage to achieve good things | Sacrificing self for others |
| A man helping his wife deliver their child | Hazardous occupations | sacrificing your life to save another |
| A man jumping on a grenade to save his fellow soldiers | help someone grab something from a high shelf | Sacrificing yourself so another may live. |
| a man shielded his wife during a shooting | helping a choking victum | Safely landing a passenger plane with no landing gear |
| A parent giving up own life to save a family member. | helping a friend | save someone from a burning building |
| a woman saved a girl from being kidnapped by pretending to be her mother | helping an elderly person | saves others |
| A woman who overcame a tough childhood adopted and raised three foster children on her own. | Helping at a car accident | Saving a baby |
| act of bravery | Helping elderly people. | saving a cat from a tree |
| Admitting mistakes | helping homeless | Saving a cat's life. |
| adopting a child in a terrible situation | Helping less fortunate people | saving a child from a burning building |
| Adopting a child in need. | Helping others | saving a child from a kidnapper |
| Adopting or taking care of a foster child | helping out at an animal shelter | saving a child from danger |
| Adoption | helping people escape a fire | SAVING A CHILD FROM DROWNING. |
| Adoption | Helping someone after a traumatic event | Saving a child from fire |
| affection | Helping someone after an accident | Saving a child's life. |
| Alek Skarlatos | Helping someone get from a abusive situation. | saving a dog from a hard life |
| all of the first responders during 911 | Helping someone in need | saving a dog from a hot car |
| always having extra tampoons if someone needs them | hero | Saving a dog's life. |
| American soldiers in battle | Holding open a door so children can escape from oncoming fire | Saving a drowning person |
| An illustrator from Colombia jumped onto subway tracks in Manhattan to help a homeless man who had fallen. | Holding the hand of a hospice patient | saving a drowning person |
| An usher confronted a gunman who opened fire at a church in Tennessee. | Hollywood Bank Heist Shootout | Saving a family from a fire. |
| Angela Pierce | Inmate firefighters prepare to battle the Rim Fire near Yosemite National Park, California | Saving a friend from an accident |
| Angela Pierce | inspiration | saving a kitten from a burning building |
| anyone standing up for what's good and kind | Intervening when it's uncomfortable | saving a life |
| Assisting the elderly | investigating someone for conspiracy | Saving a life. |
| becoming a firefighter or policeman | Jeremy Wuitschick And Johnny Wood | Saving a person from a burning building |
| Being a firefighter | Jeremy Wuitschick And Johnny Wood | Saving a person from a fire |
| Being a friefighter | Jeremy Wuitschick And Johnny Wood | saving a person from drowning |
| being a good parent | Jim Gard from Parkland High | saving a person's life |
| Being a policeman | job | Saving a person's life. |
| being a really good friend for someone with depression | joining armed forces | Saving a pet from a rescue center |
| Being in a search party | Joining the military | Saving an animal's life |
| Being kind to others. | joining the military | SAVING CHILD FROM BURNING BUILDING |
| Being selfless | Jon Meis | saving hostages |
| boldness | Jon Meis | saving lives in a storm |
| brave | jumping in front of someone to take a bullet for them | saving people out of a burning building |
| Bravery | jumping into dangerous waters to save someone drowning | saving someone drowning |
| Bringing food or medicine to the elderly or handycap | Jumping into the water to save a drowning person. | Saving someone from a burning building |
| bringing someone food | Jumping on train tracks to save fallen person. | Saving someone from a burning building |
| Bystanders dragging driver out of burning car. | Keenia Williams | Saving someone from a burning building |
| Cancer patients fighting for their lives. | Keenia Williams | Saving someone from a burning building. |
| carrying | Keeping others calm in the face of danger | saving someone from a burning car |
| catching a baby | Kyle Carpenter | Saving someone from a fire |
| catching a robber | Lauren Prezioso | Saving someone from a fire |
| child standing up for someone being bullied | Lauren Prezioso | saving someone from a fire |
| Childbirth | Lauren Prezioso | Saving someone from a heart attack |
| Children with cancer | leadership | Saving someone from a house fire |
| climbing a tree to rescue a pet | leaving a negative situation to better everyone | saving someone from a kidnapper |
| Coast Guard | Lending an ear to help comfort someone. | Saving someone from being raped or mugged. |
| Colton Haab from Parkland High | Lewis Thomas | saving someone from burning building |
| confronting a abusive spouse | Lewis Thomas | saving someone from drowning |
| Conscientious objectors. | Lewis Thomas | saving someone from drowning |
| Courage | Life sacrifice for the benefit of others. | saving someone from drowning |
| courage | Lifeguard jumping in to save someone drowing | saving someone from drowning |
| covering your loved ones with your body as a tornado hits your home | love | saving someone in a combat situation |
| Crew men restoring power in the middle of a major stom during the weekend noreaster | Man throws himself in front of active shooter to save children. | saving someone who is choking |
| danger | Man thwarts a would-be rapist. | saving someone who is choking |
| Daniel Konzelman helped the injured out of a derailed Amtrak train. | marines enlisting and going to falujia | saving someone who is drowning |
| Darnell Barton | Martin Luther King | Saving someone's life |
| Darnell Barton | Martin Luther King | saving someone's life |
| Defending an innocent person | Martin Luther King Jr. fighting for black rights | Saving someone's life |
| Defending someone from abusive authority figures. | Members of the millitary | saving someones life in any manner |
| Defending someone from harm. | Military members going into combat for America. | saving someone's life when it is not your job |
| Doctor | Military service | Schindler saving Jews |
| Doctors and nurses calmly did their jobs after a gunman stormed the hospital where they work. | Miracle on the Hudson | Search and rescue by sea |
| dodging in front of a bullet to save another person. | Moments of hope and inspiration rose above the chaos of Hurricanes Harvey and Irma. | Search and rescue in the mountains |
| Donating a kidney. | Mother Teresa's entire life | Selflessness |
| Donating an organ | MY husband staying up all night making sure my dad's oxygen tank didn't run out | serving in the army |
| donating an organ | Nelson Mandela | serving in the military |
| Donating blood | nelson mandela knowingly stayed in jail for a crime he didn't commit | Serving in the military for your country |
| donating blood | Paramedics | Shielding a person from gunfire |
| donating food to a poor family | Paramedics seeing terrible things but still helping | shooting an active shooter |
| Donating money to a needed cause | paying off a school district's lunch money debt | Soldiers |
| donating new clothes or food | people on a flight crashed the plane so the terrorists wouldn't fly it into a building | Soldiers dying for team mates - falling on explosives. |
| donating organs | People that jump into rivers to save other people | soldiers going in to protect civilians |
| donating organs or bone marrow | performing CPR | someone donating a kidney to someone |
| Donating organs to a stranger or loved one | performing cpr | someone going into a burning building to rescue someone |
| donating to cancer patients | Performing open heart surgery. | Someone performing CPR |
| donating to charity | performing the heimlich maneuver | someone pushing someone from the path of an on coming car |
| Donating to charity | Persons trying to rescue passengers from airplane downed in water. | someone running into a burning building to savesomeone |
| Donations to charity | playing it forward buying someone else a coffee | Someone stepping in front of a bullet meant for another |
| Driving safely | police catching criminals | Someone stopping a robbery or something similar by physically stopping the person. |
| Dying in the line of fire (military) | Police going into domestic violence situations. | Standing up for a friend against a bully |
| emt's | Police Officer risking her life each shift | standing up for black rights |
| emts saving a persons life | police officer risking their life to save others | standing up for equal rights |
| Entering a burning building to save some one. | Police officers | standing up for LGBT rights |
| Entering a burning building to save someone | Police officers | Standing up for what what is right |
| facing your fears for progression | Police officers confronting a shooter. | Standing up for what's right. |
| feeding a homeess person | Police Officers putting their lives on the line. | standing up to a bully |
| Feeding the homeless. | Police officers standing someone down who is threatening someone with a weapon. | standing up to a bully |
| Feeding the poor & needy | Police patrolling the streets | Standing up to a bully |
| Feeding those who are unable to feed themselves. | Policeman | standing up to politicians |
| Female inmates in California signed up to fight wildfires, at times risking their lives. | Preventing someone from committing suicide | stepping up to crime leaders |
| Fighting an hostile force | Protecting children in a school shooting. | stoping or catching a child abductor |
| Fighting for our country | protecting people in immediate danger | Stopping a bomb |
| Fighting for rights and equality of groups of people. | protecting someone being shot at | Stopping a gunman |
| Fighting for the rights of others | Protesting injustice | Stopping a murderer |
| Fighting for your country. | providing cpr to someone in need | Stopping a robbery. |
| Fighting in a war | Providing CPR to someone who is unresponsive. | Stopping a shooter. |
| Fighting in the military | pull someone from traffic | Stopping a thief |
| fighting off intruders | pulling a car from someone | Stopping an assault |
| finding a murder suspect | Pulling a child from a burning building | stopping and helping in an automobile accident. |
| Fire Rescue | pulling someone from a burning building | stranger providing cpr to a victum |
| Fire rescue | PULLING SOMEONE FROM A CAR ACCIDENT | Subway Rescue |
| Firefighter going into a burning building | pulling someone from a car accident that is leaking gas | sully sullivan saving the people on the plane |
| Firefighters | Pulling someone from a fire. | taking a bullet for someone |
| Firefighters at 9/11 | Pushing a person out of the path of a speeding car | taking a bullet for someone |
| Firefighters climbing into the Twin Towers. | Pushing someone away from oncoming car | Taking a bullet to save another |
| Firefighters entering WTC 2 on 9/11 | Putting out a fire | taking action against inequalities and unfairness |
| firefighters fighting fires | quick-thinking in a dangerous situation | taking care of someone |
| Firefighters going into a burning building to save someone. | raising your child right | talking someone out of suicide |
| Firefighters going into burning buildings. | Report crime to police | Talking someone out of suicide. |
| Firefighters on 9/11 | rescue kid from fire | Teacher jumping in front of shooter to save students in Florida school shooting |
| firefighters risked their lives to save those trapped in the rubble of 9/11 | Rescue of people on 9/11 | Teachers shielding kids from an active shooter. |
| firefighters rushing into burning buildings to help others | Rescue workers carry a child who was rescued from the rubble at the site of a collapsed residential building in Mumbai, India | Teachers teaching every day |
| firefighters saving people in burning buildings | rescueing a child from a fire | Teaching children |
| fire-fighting | rescueing a drowning victim | team player |
| Fireman | Rescuing a dog trapped in a well | Teenage girls in Nigeria, kidnapped by Boko Haram and strapped to suicide vests, managed to escape and tell their stories. |
| Fireman running into a burning house. | Rescuing a drowning baby | Temar Boggs And Chris Garcia |
| Fireman saves people from burning building. | Rescuing a kidnap victim | Temar Boggs And Chris Garcia |
| Fireman saving people from burning buildings, | rescuing someone from a bad accident | Temar Boggs and Chris Garcia chased after an abductor until he let his captive go. |
| Firemen entering burning building | rescuing someone from a burning building | The 9/11 firefighters. |
| Firemen going into burning homes to save family. | rescuing someone from a burning building | the president going to war zones to support troops |
| firemen saving people from burning buildings | rescuing someone from a fire | The President going to work with bullet proof vest. |
| firemen saving victums | Rescuing someone from a flood. | The relief workers cleaning up after a natural disaster |
| first responders going into a building not knowing the situation | Rescuing someone from a wreck | Treating everyone as equals |
| Getting someone out of a wrecked car | rescuing someone from drowning | Two teenage boys are being hailed as heroes after they chased a car carrying a kidnapped girl on their bicycles. |
| giving a home to a shelter animal | Resuscitating someone | volunteering |
| giving CPR to a person that needs it | Risking your life to save another. | Volunteering as an EMT |
| Giving food to a homeless person. | Robert Mohr And Rodney Lindley | volunteering at a soup kitchen |
| Giving money to people who need it. | Robert Mohr And Rodney Lindley | Volunteering at a soup kitchen |
| Giving money to someone in need. | Rosa Parks | Volunteering your free time to those in need |
| Giving someone an interest free loan when they are poor | running into a burning building | Washington crossing the Delaware |
| giving someone cpr | Running into a burning building to save someone | Whistle-blowers. |
| Giving to charity | running into a burning building to save someone | Workers going into radioative nuclear factory in Japan |
| giving your life | Running into a fire to save someone | Working overseas for charity |
| Going into a burning building to rescue people. | running off a stalker | working with disadvantaged youths |
| going into the ocean to save someone from drowning | Rushing into a burning building | young child protecting mother from abuse |
| Going out on the ice to rescue a person who went thru the ice. | Rushing into a burning building after a child |  |


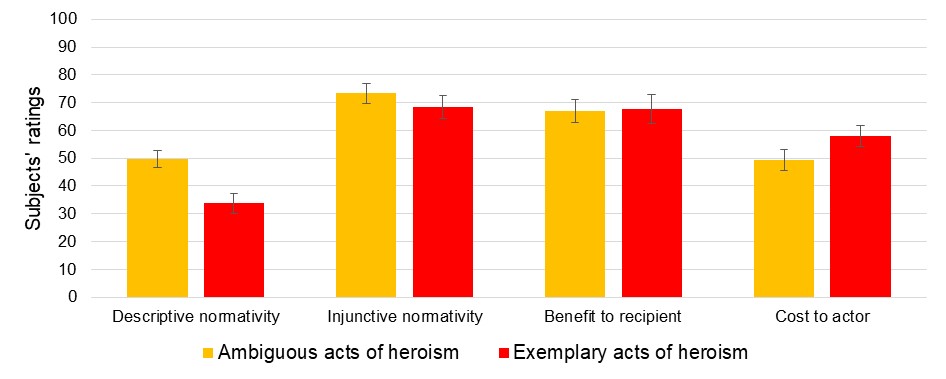


**SI Figure 1. Exemplary acts of heroism are seen as *rarer* and *more costly* to actors (though *not* more beneficial to recipients) than ambiguous acts of heroism.** Shown are means (with 95% CIs) of subjects’ ratings on four measures (0-100 slider scales; see SI Fig. 2 for distributions) aggregated over the 5 exemplary and 5 ambiguous acts of heroism.


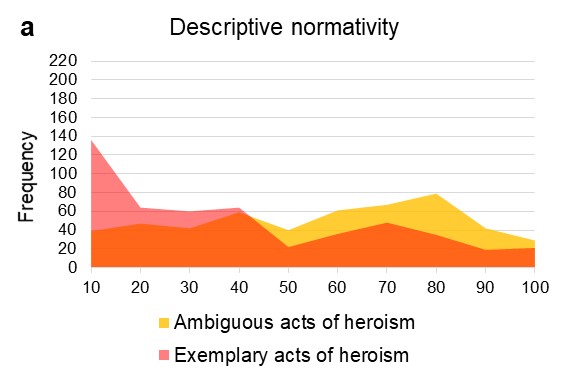

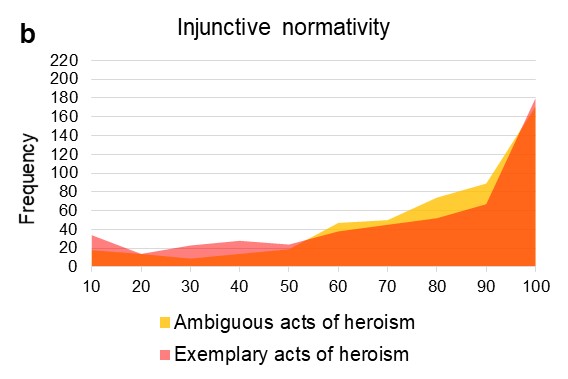


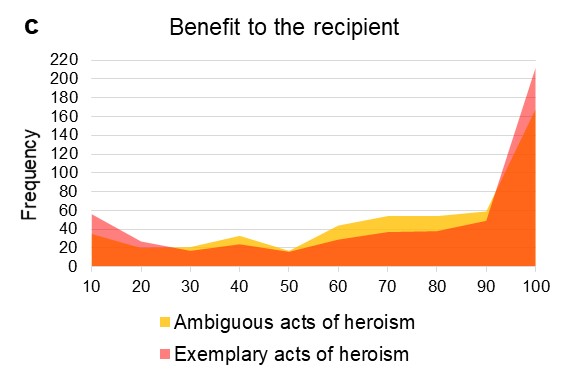

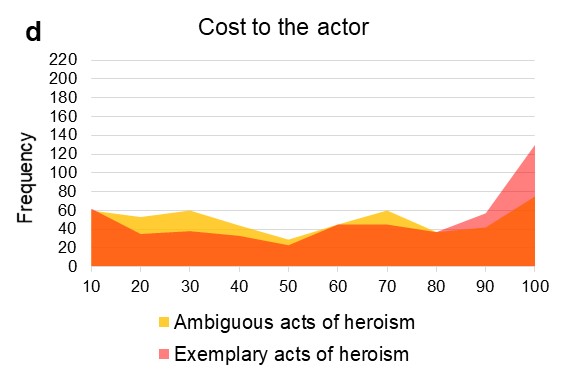


**SI Figure 2. Subjects’ perceptions of exemplary v. ambiguous acts of heroism.** Shown are distributions of subjects’ ratings on four measures (0-100 slider scales): (**a**) *descriptive normativity* (“In your opinion, how many people in your community do this behavior?”); (**b**) *injunctive normativity* (“In your opinion, how much do people in your community think doing this behavior is what you are supposed to do?”); (**c**) *benefit to the recipient* (“In your opinion, how much benefit (in terms of money, time, effort, etc.) does the recipient of this behavior receive?”); and (**d**) *cost to the actor* (“In your opinion, how much cost (in terms of money, time, effort, etc.) does the person who does this behavior incur?”).


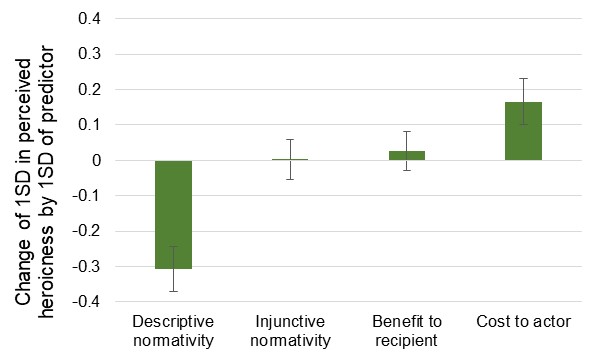


**SI Figure 3. More heroic acts are seen as *rarer* and *more costly* to actors (though *not* more beneficial to recipients nor less obligatory) than ambiguous acts of heroism.** Shown are standardized coefficients (with 95% CIs) of subjects’ ratings on four measures (0-100 slider scales) predicting the heroicness of the acts as measured on a continuous scale (from Study 2).

**SI Table 2. Robustness check of Study 3 regression results.** Results from Study 3 are robust to conducting regression analyses as OLS regression with proportion of Study 2 participants indicating the behavior was heroic (standardized) as the independent variable or a logistic regression with a classification of behavior as exemplary v. ambiguous acts of heroism as a binary independent variable.


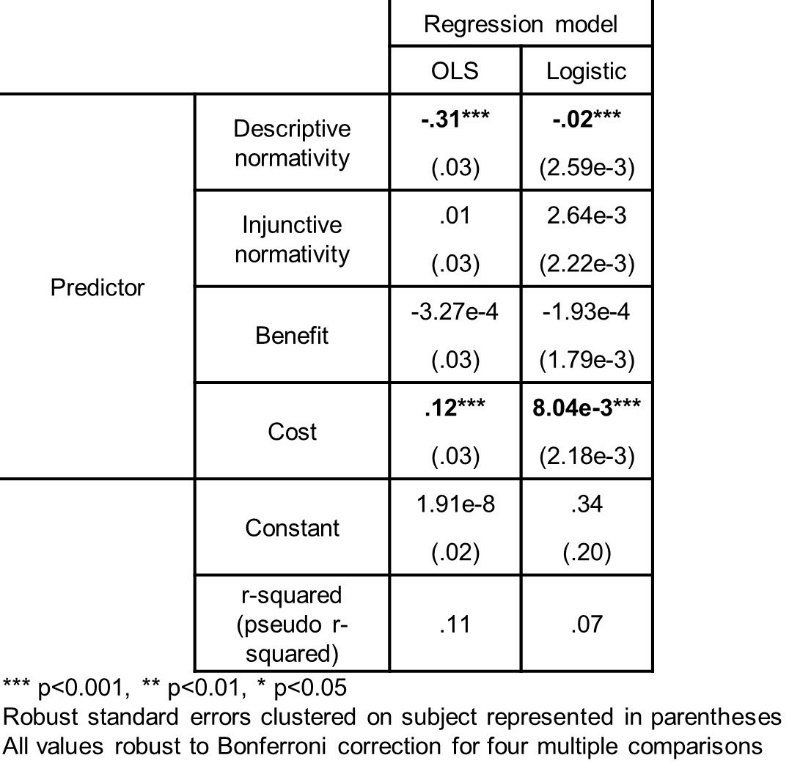

Supplement: Supplementary file 1 [file Data_Sheet_1.docx]
